# Combined Antiviral and Cytoprotective Action of Rosmarinic Acid Against EV-A71 Infection: A Potential Therapeutic Strategy

**DOI:** 10.3390/pathogens14070622

**Published:** 2025-06-23

**Authors:** Junping Lv, Weishi Lin, Siqi Chao, Jing Xie, Yue Cao, Jinfeng Tie, Yuehua Ke, Binan Lu, Zongran Pang

**Affiliations:** 1 School of Pharmacy, Minzu University of China, Beijing 100081, China; 2 Key Laboratory of Ethnomedicine, Minzu University of China, Ministry of Education, Beijing 100081, China; 3 Center for Disease Control and Prevention of PLA, Beijing 100071, China; 4 Department of Bacteriology, Capital Institute of Pediatrics, 2 Yabao Road, Beijing 100020, China

**Keywords:** cell damage, Enterovirus A71, inflammatory response, rosmarinic acid, viral replication

## Abstract

Enterovirus A71 (EV-A71), a major etiological agent of hand-foot-mouth disease, can cause severe neurological complications. However, the mechanisms underlying EV-A71-induced cell damage and potential therapeutic strategies remain inadequately understood. Here, we investigated EV-A71 replication dynamics and associated cytopathic effects in nine distinct cell lines, including epithelial, neuronal, immune, and other cell types. Cell viability, membrane integrity, and energy metabolism were assessed using Cell Counting Kit-8 (CCK-8), lactate dehydrogenase (LDH), and adenosine triphosphate (ATP) assays. The antiviral activity of rosmarinic acid (RA), a natural polyphenol, was evaluated by plaque reduction, qPCR, and Western blot. EV-A71 exhibited cell-type-specific replication and cytotoxicity patterns. RA significantly preserved cell viability, reduced LDH release, maintained ATP levels, and suppressed IL-6 expression. Mechanistically, RA inhibited viral replication by downregulating VP1 expression and viral RNA levels. Molecular docking indicated strong binding of RA to the hydrophobic pocket of VP1, potentially disrupting virus-host interactions. Collectively, these findings highlight RA’s combined antiviral and cytoprotective potential, supporting its candidacy as a therapeutic agent against EV-A71 infection.

## 1. Introduction

Enterovirus 71 (EV-A71), a predominant viral pathogen of hand, foot, and mouth disease (HFMD), poses a significant global public health challenge due to its neurotropism, which can lead to severe complications such as encephalitis and acute flaccid paralysis [1]. Despite the availability of an inactivated EV-A71 vaccine, its low vaccination coverage and limited medium- to long-term efficacy in the Asia-Pacific region leave a considerable immunoprotection gap in high-risk populations [2,3]. Additionally, the lack of specific antiviral drugs remains the mainstay of clinical management for managing severe clinical cases. Ribavirin (RBV), one of the few antiviral drugs in use, is limited by dose-dependent cytotoxicity and teratogenic risks [4,5,6,7]. Furthermore, most in vitro studies on EV-A71 rely on single-cell models (e.g., Vero or RD cells), which may not adequately reflect the complexity of host–virus interactions observed in vivo.

The host specificity of EV-A71 is shaped by multiple factors, including heterogeneity in receptor expression (e.g., SCARB2 and PSGL-1), thresholds for innate immune activation, and the metabolic microenvironment [8,9,10]. However, comprehensive investigations examining viral replication efficiency across diverse cell types and their corresponding mechanisms of virus-induced cytotoxicity remain limited. Addressing this gap is essential for identifying conserved therapeutic targets to combat EV-A71-induced pathology.

Natural phenolic acids have attracted considerable attention for their broad-spectrum antiviral properties and relatively low cytotoxicity, making them promising candidates for therapeutic development [11,12]. RA, a natural polyphenolic compound primarily derived from plants of the *Lamiaceae* family, has been reported to inhibit the replication of various viruses, including influenza and herpes simplex virus (HSV), by modulating viral entry, replication, and host immune responses [13,14]. Preliminary studies also suggest that RA may interfere with viral RNA synthesis and regulate cellular antiviral pathways; however, its antiviral activity against EV-A71 remains poorly characterized.

In this study, we first examined the tissue-specific replication dynamics of EV-A71 and the associated cytopathic effects across diverse cell lines representing epithelial, neuronal, immune, and other types. Subsequently, we evaluated the antiviral efficacy of Rosmarinic Acid (RA) in a representative model and found that RA effectively mitigated EV-A71-induced cytotoxicity, preserved cellular function, and suppressed viral replication and inflammation, providing mechanistic insight into its protective role.

## 2. Materials and Methods

### 2.1. Compounds

Rosmarinic acid (CAS No. 20283-92-5) and ribavirin (CAS No. 36791-04-5), each with a purity of ≥98%, were purchased from Chengdu Alfa Biotechnology Co., Ltd. (Chengdu, China). For experimental use, both compounds were freshly dissolved in a complete cell culture medium consisting of DMEM supplemented with 10% fetal bovine serum (FBS). The MOCK group was established by adding the same volume of complete medium without drug treatment or virus infection.

### 2.2. Cell Culture and Viral Preparation

Nine distinct cell lines, including HeLa (cervical carcinoma), RD (rhabdomyosarcoma), HUVEC (umbilical vein endothelial), Hep-2 (laryngeal epidermoid carcinoma), Vero (African green monkey kidney), BHK (Syrian hamster kidney), NSC34 (motor neuron-like), bEnd.3 (brain microvascular endothelial), and RAW264.7 (monocyte/macrophage leukemia) were used in this study. All cell lines were preserved and provided by the PLA Center for Disease Control and Prevention (PLA CDC, Beijing, China). Cells were maintained in DMEM (Gibco, Grand Island, NY, USA, #C11965500BT) supplemented with 10% fetal bovine serum (Gibco, Grand Island, NY, USA, #10091148) at 37 °C in a 5% CO_2_ atmosphere.

The EV-A71 viral strain was also obtained from the PLA CDC. Virus propagation was performed in Vero cells, and viral titers were determined using a TCID_50_ assay with eight replicates per dilution in 96-well plates. Viral stocks were aliquoted and stored at −80 °C until further use.

### 2.3. Measurement of Cytotoxicity and Cell Viability

The cytotoxic effects of EV-A71 infection on host cell lines were assessed under a range of multiplicities of infection (MOIs, 0.5–10; *n* = 6). Cell metabolic activity was evaluated using the Cell Counting Kit-8 (CCK-8; Yeasen, Shanghai, China, #40203ES60). At 48 h post-infection, 10 μL of CCK-8 reagent was added to each well of a 96-well plate, followed by incubation at 37 °C for 1 h. Absorbance was then measured at 450 nm using a microplate reader.

Cell membrane integrity was determined by lactate dehydrogenase (LDH) release using a commercial LDH assay kit (Beyotime, Shanghai, China, #C0017). A total of 100 μL of culture supernatant was mixed with catalyst solution (1:45), incubated in the dark for 30 min, and measured at dual wavelengths of 490/630 nm.

Intracellular ATP levels were quantified using the CellTiter-Glo Luminescent Cell Viability Assay (Promega, Madison, WI, USA, #G7570). Equal volumes of reagent and cell lysate were mixed, followed by orbital shaking for 10 min at room temperature. Luminescence was then measured using a multimode plate reader.

### 2.4. Antiviral Studies of RA

The CCK8 assay was used to detect the cytotoxicity of RA and RBV to determine their maximum non-toxic concentrations, which were set as the maximum dosing concentrations, and a series of concentration gradients were set based on this. HeLa cells were seeded in 12-well plates and cultured overnight under standard conditions (37 °C, 5% CO_2_). The medium was then aspirated, and an EV-A71 virus solution with an MOI of 0.5 and a drug-containing medium were added simultaneously. After 48 h of culture, cell samples were collected. mRNA levels were detected by qPCR, and protein expression was measured by Western blot.

A multiplicity of infection (MOI) of 0.5 was selected for antiviral evaluation to ensure measurable drug efficacy while avoiding excessive cytopathic effects. At a higher MOI (e.g., MOI = 1), rapid and widespread cell lysis in virus-only groups may lead to massive viral particle release, which can obscure the protective effects of antiviral compounds.

### 2.5. Western Blot Analysis

Cellular proteins were extracted using RIPA buffer (Beyotime, Shanghai, China, #P0013B) containing a protease inhibitor cocktail (Beyotime, Shanghai, China, #ST506). After quantification by BCA assay, 20 μg protein/sample was separated on 12% SDS-PAGE and transferred to PVDF membranes. Blocking with 5% skim milk in TBST preceded incubation with primary antibodies: anti-VP1 (custom antibody, 1:3000) and anti-GAPDH (CST, Danvers, MA, USA, #97166S, 1:5000) at 4 °C overnight.

HRP-conjugated secondary antibodies (CST, Danvers, MA, USA, #7076S) were applied at 1:3000 for 1 h at 25 °C. Signals were developed with ECL Prime (Thermo, Waltham, MA, USA, #32132) and imaged using Amersham Imager 800 (Cytiva, Marlborough, MA, USA). GAPDH-normalized band intensities were analyzed with ImageJ (Version 1.48V) software.

### 2.6. Plaque Assay for Viral Titration

Viral titers pre- and post-treatment were determined through standard plaque assays. Viral supernatant was serially diluted 10-fold in serum-free medium, then used to infect 90% confluent Vero cell monolayers. Specifically, cells in 12-well plates were inoculated with 200 μL of a 10^−3^ diluted virus suspension at 37 °C for 1 h, with periodic rocking. Following the removal of unadsorbed viral particles, RA and RBV were diluted to the desired twofold concentrations in DMEM containing 4% FBS and then mixed in a 1:1 ratio with 4% low-melting-point agarose. The resulting final overlay contained RA (15.6–62.5 μM) or RBV (15.6 μM, as a positive control), 2% agarose, and 2% FBS, providing optimal conditions for cell maintenance. Parallel control wells received identical overlay medium without drug supplementation. After 72 h incubation at 5% CO_2_, plaques were visualized by crystal violet staining [15] (Solarbio, Beijing, China, #G1065).

### 2.7. Quantitative PCR

Total RNA from EV-A71-infected cells was extracted using the Tiangen Viral RNA Kit (DP315, China) according to the manufacturer’s instructions and eluted in 60 μL of RNase-free water. Reverse transcription was performed using 1 μg of RNA and the PrimeScript RT Kit (Tiangen, Beijing, China, #KR116).

SYBR Green-based qPCR was used to quantify cytokine mRNA levels (IL-6, IL-1β, TNF-α), while TaqMan probe-based qPCR was used to quantify viral RNA.

The primer sequences were as follows (Table 1):

### 2.8. TCID_50_ Assay

Following the 48 h antiviral treatment in HeLa cells at an MOI of 0.5, the culture supernatant and cells were subjected to three freeze–thaw cycles to release intracellular virus particles. The lysates were then serially diluted 10-fold and inoculated into 96-well plates pre-seeded with confluent RD cells. Each dilution was tested in six replicates. After 72 h of incubation at 37 °C with 5% CO_2_, cytopathic effects (CPE) were evaluated, and TCID_50_ values were calculated using the Reed–Muench method.

### 2.9. Molecular Docking Analysis

The protein structure was obtained from the Protein Data Bank (PDB), and the structure of RA was retrieved from the PubChem database. The EV-A71 VP1 subunit was extracted from the crystal structure (PDB ID: 3VBS) using PyMOL v2.5.4. Water molecules and non-protein ligands were removed, and hydrogens were added to optimize the protein structure. The extracted VP1 subunit was saved as a PDB file for subsequent docking. VP1 is the major surface-exposed structural protein of EV-A71 and plays a crucial role in receptor recognition and viral entry. Therefore, VP1 was selected as the primary target for molecular docking studies to explore potential antiviral binding sites of rosmarinic acid.

Blind docking of RA to VP1 was performed using the CB-Dock2 online server. This tool predicts the ligand-binding cavity based on protein topology and energy scoring. The conformation with the lowest energy score (−9.1) was selected for subsequent analysis. Visualization analysis was carried out using PyMOL.

### 2.10. Statistical Analysis

All statistical analyses were performed using GraphPad Prism version 8.0 (GraphPad Software Inc., San Diego, CA, USA). Comparisons among multiple groups were conducted using one-way analysis of variance (ANOVA), followed by Tukey’s multiple comparisons test when a significant overall effect was observed (*p* < 0.05). Data are expressed as mean ± standard deviation (SD). Viral titers (TCID50) were log-transformed prior to analysis. Statistical significance was defined as * *p* < 0.05, ** *p* < 0.01, and *** *p* < 0.001.

## 3. Results

### 3.1. The Multiplication Rates of EV-A71 Infection Vary Greatly in Different Host Cells

**Figure 1:**
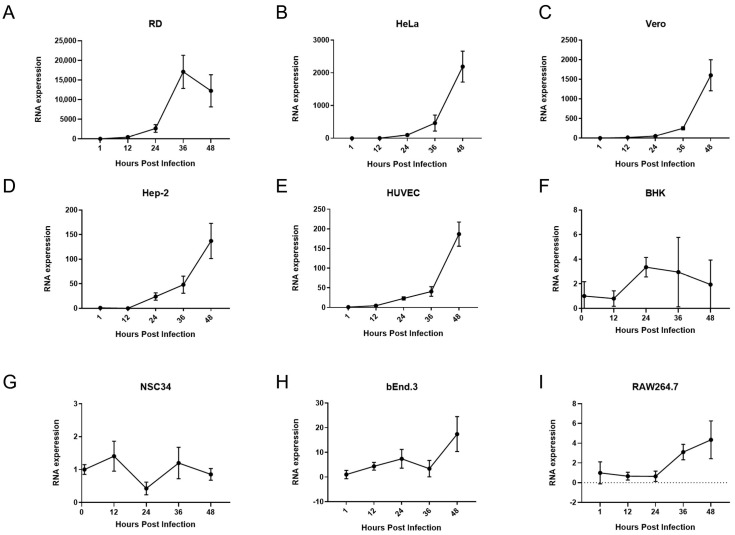
Rate of multiplication of EV-A71 infection in multiple host cell lines. Intracellular EV-A71 mRNA levels were quantified in (**A**) RD, (**B**) HeLa, (**C**) Vero, (**D**) HUVEC, (**E**) Hep-2, (**F**) BHK, (**G**) NSC34, (**H**) bEnd.3, and (**I**) RAW264.7 cells at 1, 12, 24, 36, and 48 h post-infection (hpi). Cells were seeded in 24-well plates and infected with EV-A71 at a multiplicity of infection (MOI) of 1. Total RNA was extracted at each time point, and viral RNA levels were measured by quantitative real-time PCR (qPCR). Relative viral RNA abundance was calculated using the 2^−ΔΔCt^ method, normalized to GAPDH expression and compared to MOCK (uninfected control) cells (*n* = 4).

The replication kinetics of EV-A71 varied markedly among the tested cell lines. Robust viral replication was observed in RD, HeLa, Vero, HUVEC, and Hep-2 cells, suggesting that these cell types provide a permissive environment for EV-A71 proliferation (Figure 1). In contrast, BHK, NSC34, bEnd.3, and RAW264.7 cells supported only limited viral replication, indicating a relatively restrictive cellular context. These findings are consistent with previous reports demonstrating that host cell type and its intrinsic molecular machinery critically influence viral replication dynamics [16].

### 3.2. Impacts of EV-A71 Infection on the Viability of Host Cells

The cytotoxicity of EV-A71 infection was assessed by measuring LDH release, intracellular ATP levels, and cell viability via CCK-8 assays. Results revealed distinct cell-type-specific responses.

**Figure 2:**
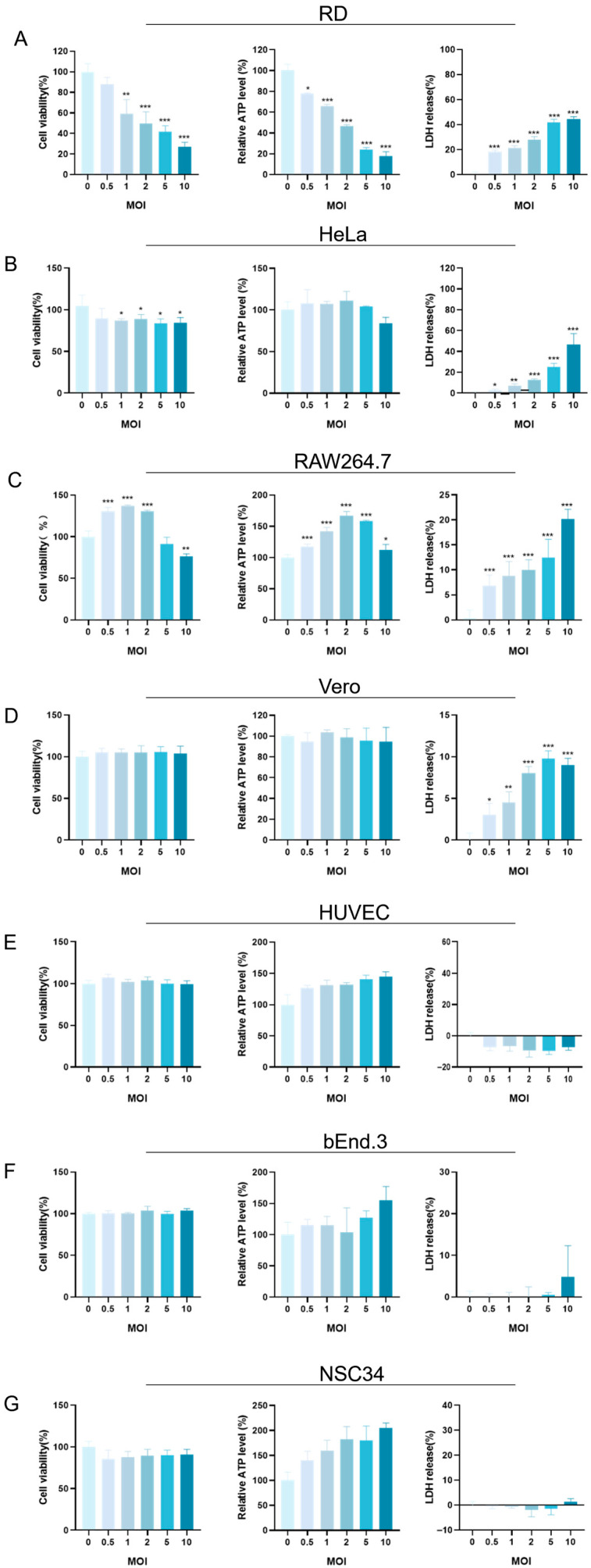
Effects of EV-A71 infection on host cell viability and cytotoxicity across multiple MOI gradients. (**A**) RD, (**B**) HeLa, (**C**) RAW264.7, (**D**) Vero, (**E**) HUVEC, (**F**) bEnd.3, and (**G**) NSC34 cells were infected with EV-A71 at increasing multiplicities of infection (MOI 0, 0.5, 1, 2, 5, and 10). The MOI = 0 group received only virus dilution buffer and served as the uninfected control. After 48 h, cell viability and damage were assessed using the CCK-8 assay, intracellular ATP quantification, and LDH release assay. Panels A–G present the respective results for each cell line across different MOI conditions. Data are expressed as mean ± SD of three independent experiments. Statistical significance was evaluated using one-way ANOVA. * *p* < 0.05, ** *p* < 0.01, *** *p* < 0.001.

In RD cells, an increasing MOI led to a marked reduction in cell viability, accompanied by decreased ATP levels and elevated LDH release. Notably, when the MOI increased to 5 or 10, metabolic activity was significantly suppressed and membrane integrity was compromised, indicating severe cellular damage (Figure 2A).

Interestingly, RAW264.7 cells exhibited a biphasic response. At a low MOI, both ATP levels and cell viability slightly increased, suggesting a transient enhancement in metabolic activity. However, at a higher MOI (≥5), this trend reversed: ATP levels and viability declined, while LDH release increased, reflecting progressive cytopathic effects (Figure 2C).

In the case of HeLa cells, although the changes in CCK-8 and ATP levels were relatively small at a lower MOI, a significant increase in LDH release was detected at a high MOI. When the MOI reached 2 or higher, especially at 5 and 10, the LDH release showed a sharp increase, suggesting that the cell membrane of HeLa cells was increasingly damaged as the infection progressed (Figure 2B).

Vero, HUVEC, bEnd.3, and NSC34 cells also showed specific responses (Figure 2D–G). Overall, their cell viability and ATP levels did not change drastically, and the LDH release remained relatively stable under most MOI conditions. However, Vero cells exhibited a slight increase in LDH release at a high MOI. When the MOI reached 10, a small but observable increase in LDH release occurred, indicating that the cell membrane of Vero cells might be experiencing some degree of disruption at high-infection-intensity conditions.

In summary, EV-A71 induced variable cytotoxic effects across different host cells, with severity modulated by both the infection dose and intrinsic cellular characteristics.

### 3.3. Rosmarinic Acid Mitigates EV-A71-Induced Cytotoxicity and Enhances Host Cell Viability

**Figure 3:**
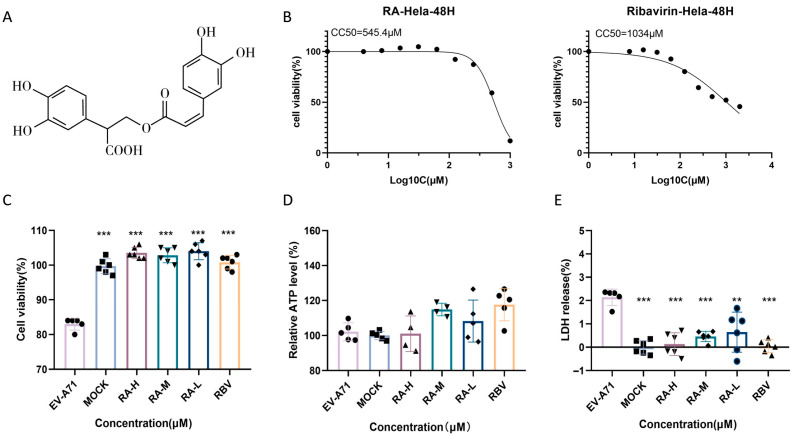
Rosmarinic acid improves the viability of infected cells. (**A**) Chemical structure of rosmarinic acid (RA). (**B**) Cytotoxicity curves for RA and ribavirin (RBV) in HeLa cells after 48 h treatment. Compounds were tested in twofold serial dilutions to determine the half-maximal cytotoxic concentration (CC_50_). Data are presented as mean ± SD (*n* = 6). (**C**–**E**) Experimental overview: HeLa cells were infected with EV-A71 (MOI = 1) and treated simultaneously with graded concentrations of RA (RA-H: 62.5 µM; RA-M: 31.3 µM; RA-L: 15.6 µM) or RBV (15.6 µM). MOCK refers to the uninfected, untreated group. Cell viability was assessed via CCK-8, intracellular ATP levels, and LDH release after 48 h. ** *p* < 0.01, *** *p* < 0.001.

RA, a natural polyphenolic compound abundant in *Lamiaceae* plants and structurally depicted in Figure 3A, was evaluated for its cytotoxicity and protective potential in EV-A71-infected HeLa cells. Cytotoxicity curves revealed that RA exhibited a significantly higher half-maximal cytotoxic concentration (CC_50_) than RBV, indicating its lower cytotoxicity at elevated concentrations. Based on these results, 62.5 μM RA and 15.6 μM RBV were identified as their respective maximum non-toxic concentrations and were thus used in subsequent assays (Figure 3B).

The protective effect of RA under EV-A71 infection was further evaluated in HeLa cells. CCK-8 assay results (Figure 3C) indicated a significant reduction in cell viability upon viral infection, which was substantially restored by RA treatment in a dose-dependent manner. Treatment with 62.5, 31.3, or 15.6 μM RA restored viability close to baseline levels, demonstrating robust cytoprotective effects. RBV at 15.6 μM exhibited similar efficacy.

Intracellular ATP levels (Figure 3D) were markedly reduced in virus-infected cells, reflecting impaired metabolic activity. RA treatment significantly increased ATP production, suggesting an improvement in cellular energy status. Similarly, LDH release (Figure 3E), an indicator of membrane damage, was elevated following infection but was significantly reduced by RA at all tested concentrations. These findings indicate that RA effectively mitigates EV-A71-induced cytotoxicity by preserving viability, enhancing energy metabolism, and maintaining membrane integrity.

### 3.4. Rosmarinic Acid Modulates the Expression of Inflammatory Cytokines

**Figure 4:**
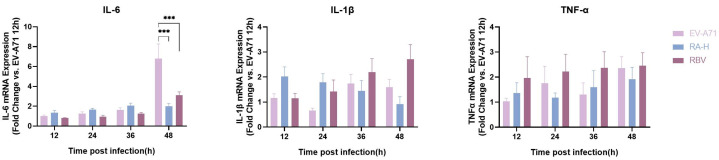
Time-dependent effects of RA on mRNA expression of inflammatory cytokines in EV-A71-infected cells. qPCR analysis of IL-6, IL-1β, and TNF-α mRNA expression at 12, 24, 36, and 48 h post-infection (hpi). Cells were infected with EV-A71 at a multiplicity of infection (MOI) of 0.5 and treated with high-dose RA (RA-H: 62.5 µM) or ribavirin (RBV: 15.6 µM). mRNA levels were normalized to GAPDH and calibrated against the MOCK group (uninfected and untreated), which was set to 1. Data are presented as bar graphs (mean ± SD, *n* = 6) for each cytokine and time point. Statistical significance was assessed by one-way ANOVA; *** *p* < 0.001.

The expression of inflammatory cytokines IL-6, IL-1β, and TNF-α in EV-A71-infected cells treated with RA and RBV was analyzed using qPCR (Figure 4). At 48 h post-infection, IL-6 mRNA levels in the EV-A71 group were significantly higher than those in the RA- and RBV-treated groups (*p* < 0.05). However, no significant regulation of IL-6 expression was observed at early time points (12, 24, or 36 hpi). For IL-1β, no consistent regulation of IL-1β mRNA expression was observed at any time point across groups. Similarly, TNF-α levels showed no notable changes in response to RA or RBV treatment. These results indicate that RA treatment effectively reduces EV-A71-induced IL-6 expression but has minimal impact on IL-1β and TNF-α levels.

### 3.5. Rosmarinic Acid Exhibits Antiviral Efficacy In Vitro

**Figure 5:**
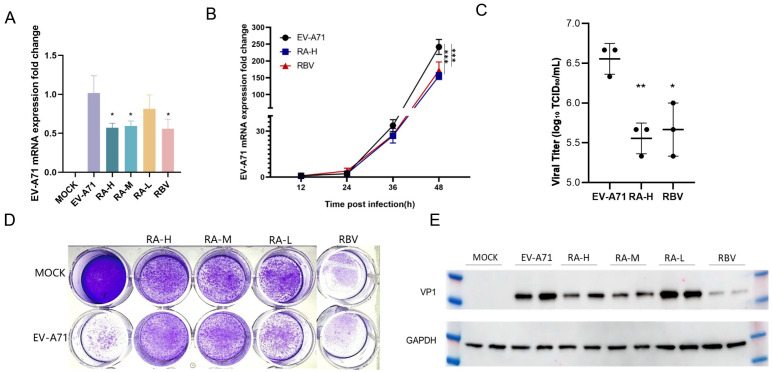
Effects of rosmarinic acid on EV-A71 viral RNA, protein expression, and plaque formation. (**A**) qPCR analysis of EV-A71 viral RNA levels in HeLa cells 48 h post-infection following treatment with RA-H (62.5 µM), RA-M (31.3 µM), RA-L (15.6 µM), or RBV (15.6 µM). MOCK: uninfected, untreated control. EV-A71: infected, untreated group. Data are expressed as mean ± SD (*n* = 6). (**B**) Time-course analysis of EV-A71 mRNA expression at 12, 24, 36, and 48 hpi in HeLa cells treated with RA-H or RBV. EV-A71 group: black circles; RA-H group: blue squares; RBV group: red triangles (*n* = 3). (**C**) Viral titers in the infected HeLa cells treated with RA-H or RBV were measured by TCID_50_ assay in RD cells at 48 hpi. Results are shown as mean ± SD (*n* = 3). (**D**) Plaque reduction assay performed in Vero cells. Cells were infected with EV-A71 and overlaid with agar containing RA (62.5, 31.3, or 15.6 µM) or RBV (15.6 µM). Both RA and RBV treatments led to reduced plaque formation. (**E**) Western blot analysis of VP1 protein levels in HeLa cells 48 h post-infection and treatment. GAPDH served as the loading control. All experiments were conducted at MOI = 0.5. Statistical significance was determined by one-way ANOVA: * *p* < 0.05, ** *p* < 0.01, *** *p* < 0.001 vs. EV-A71 group.

qPCR analysis demonstrated that RA reduced EV-A71 viral mRNA levels in a dose-dependent manner (Figure 5A). RA-H showed the strongest inhibitory effect. For example, compared to the untreated EV-A71-infected group, the viral mRNA levels decreased to approximately 0.57-fold in the RA-H treated group. RA-M also had a significant inhibitory effect with similar efficacy. RA-L had weaker effects in reducing viral mRNA levels. RBV, the positive control, also exhibited significant inhibition. Additionally, at all-time points, the viral load in the RA-H treatment group was lower than that in the EV-A71-infected group. Moreover, a significant difference was observed at 48 h (Figure 5B).

Western blot analysis confirmed that RA decreased the expression of EV-A71 viral protein VP1 in a dose-dependent manner (Figure 5E). High and medium doses showed comparable inhibition. For instance, the intensity of the VP1 protein band in the 62.5 µM and 31.3 µM RA-treated groups was significantly lower than that in the EV-A71-infected group. Lower doses had weaker effects. The positive control RBV also suppressed VP1 expression.

We conducted plaque reduction assays using Vero cells to evaluate the impact of RA and RBV on viral plaque formation (Figure 5D). The MOCK group, which received neither virus nor drug treatment, served as the uninfected baseline control, while the EV-A71 group was infected with the virus alone. Treatment groups with different concentrations of RA (62.5, 31.3, 15.6 μM) and RBV (15.6 μM) showed varying degrees of changes in viral plaques. The number of plaques in the RA-treated groups was significantly reduced compared to the EV-A71-infected group.

To comprehensively assess the changes in viral titers, after adding different concentrations of high-dose RA (RA-H) and RBV to EV-A71-infected cells, we measured the 50% tissue culture infectious dose (TCID50) using RD cells (Figure 5C). Different from the previous detection of viral mRNA levels by qPCR, the TCID50 assay directly reflects the infectivity of live viruses, which is defined as the amount of virus capable of causing infection in half of the cell cultures. Judging from the experimental results, the trends of TCID50 data in different treatment groups corroborate the results of viral mRNA level detection by qPCR. After the addition of RA-H and RBV, the TCID50 values decreased significantly, indicating that the infectivity of the virus was inhibited. This further confirms that RA can effectively inhibit the replication of EV-A71 in vitro.

### 3.6. Rosmarinic Acid Exhibits Strong Binding Affinity to EV-A71 VP1

**Figure 6:**
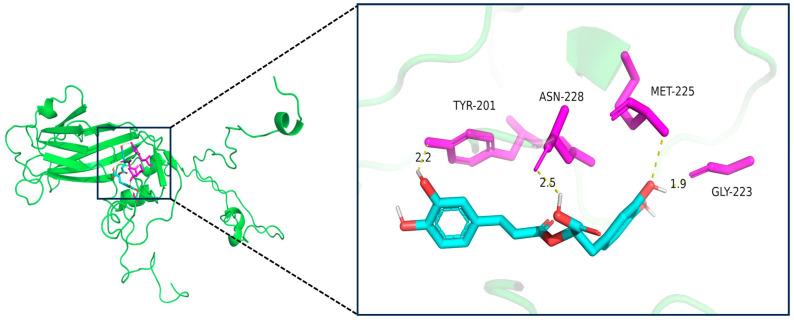
Binding mode of RA with key residues in the binding pocket of EV-A71 VP1 protein. The left panel shows the overall 3D structure of the VP1 protein (green) with the ligand-binding region highlighted. The right panel provides a magnified view of the binding pocket, illustrating the interaction between rosmarinic acid (RA, cyan) and key residues TYR-201, ASN-228, MET-225, and GLY-223 (magenta). Hydrogen bonds are shown as dashed lines, and their bond lengths (in Å) are indicated. RA forms three hydrogen bonds with VP1, and the interaction with MET-225 is hydrophobic in nature, with a distance of 3.8 Å, exceeding the conventional hydrogen bond threshold. Therefore, this interaction is classified as hydrophobic rather than a hydrogen bond.

Molecular docking analysis revealed that RA binds to the hydrophobic cavity of the EV-A71 VP1 protein within pocket C1, exhibiting a high-affinity binding with a Vina score of −9.1 (Table 2), which exceeds the high-affinity threshold (−7.0) established for AutoDock Vina-based scoring and indicates a robust binding mode consistent with benchmark validation [17,18,19]. Structural analysis showed that RA forms three hydrogen bonds with key residues in the binding pocket: a 1.9 Å bond with the backbone carbonyl group of GLY-223, a 2.2 Å bond with the side chain of TYR-201, and a 2.5 Å bond with ASN-228. Additionally, RA interacts hydrophobically with MET-225 (Figure 6). The C1 pocket has a volume of 2739 Å^3^, providing ample space for RA binding. In comparison, other pockets (C2, C3, C4, C5) exhibited lower Vina scores ranging from −5.9 to −5.6, with smaller cavity volumes, making C1 the most favorable binding site for RA (Table 2). The favorable binding affinity and interactions observed between RA and pocket C1 of VP1 highlight a possible structural basis for its antiviral activity.

## 4. Discussion

In this study, we investigated the replication characteristics of EV-A71 in several representative host cell lines to gain insights into its host range and replication mechanisms. Our findings demonstrate that RA exhibits a dose-dependent protective effect by inhibiting EV-A71 replication in vitro and alleviating virus-induced cytopathic effects. These results suggest that RA may serve as a promising antiviral agent for reducing EV-A71-mediated cellular damage. Notably, our observations are consistent with previous studies highlighting the antiviral potential of RA, particularly against RNA viruses [20].

A key observation in our study was the variability in EV-A71 replication across different host cell types. In line with previous in vivo studies that highlighted differences in EV-A71 replication rates across various organs, our in vitro results further emphasize that the biological characteristics of host cells—such as the expression levels of surface receptors and the intracellular environment—play critical roles in viral replication efficiency [16,21,22]. These variations not only impact the virus’s ability to spread within the host but also influence the severity of clinical symptoms observed in patients [23]. Understanding the molecular underpinnings of this tissue-specific replication is essential for developing targeted therapeutic strategies and improving antiviral treatments.

RA, a phenolic compound abundant in *Lamiaceae* plants, has garnered attention for its broad-spectrum antiviral properties. Previous studies have demonstrated the antiviral activity of *Lamiaceae* plant extracts against various RNA and DNA viruses, with RA likely being a key active component [20,24,25]. Our results suggest that RA may offer potential advantages over conventional antiviral agents, such as RBV. Specifically, RA exhibits lower cytotoxicity, which makes it a potentially safer alternative for long-term antiviral therapy. Additionally, the natural abundance of RA makes it a cost-effective candidate for development into a widely accessible antiviral treatment. However, further research is needed to investigate the detailed mechanisms behind its antiviral effects and assess its efficacy in vivo and in clinical settings.

In addition to its direct antiviral activity, RA also appears to modulate host inflammatory responses. Specifically, RA significantly reduced IL-6 expression at later stages of infection, suggesting that it could mitigate virus-induced pro-inflammatory damage. In contrast, IL-1β and TNF-α expression did not show consistent regulatory patterns, highlighting the complexity of RA’s effects on host immune responses. This observation points to the need for future research to explore how RA impacts other aspects of the host immune system and its potential role in modulating inflammatory cytokines during viral infection.

The molecular docking analysis further strengthens our understanding of RA’s antiviral mechanism. Our results indicate that RA binds with high affinity to the hydrophobic cavity of the EV-A71 VP1 protein, particularly within pocket C1. The Vina score of −9.1 suggests a stable binding interaction, potentially disrupting VP1’s function. Since VP1 plays a crucial role in recognizing and binding to host cell receptors during the early stages of infection, RA’s binding to this site could prevent the virus from attaching to host cells [26]. This hypothesis is supported by our in vitro data, which show that RA significantly inhibits EV-A71 replication. The formation of hydrogen bonds between RA and key residues of VP1, coupled with hydrophobic interactions, may cause conformational changes in the VP1 protein, disrupting the assembly of the viral capsid and thereby hindering viral replication. This mechanism is consistent with previously reported small-molecule-mediated inhibition of viral capsid assembly, such as the disruption of HIV-1 capsid function by targeted compounds [27]. These findings provide a molecular basis for RA’s antiviral activity and suggest that its interaction with VP1 may be a key factor in its efficacy. Compared to RA, ribavirin (RBV) exhibits weaker binding affinity to VP1 (Vina score: −6.7) and fails to form stable hydrogen bonds within the core binding region (Supplementary Data S1). According to previous studies, its antiviral activity primarily relies on inhibiting viral RNA polymerase and depleting intracellular nucleotide pools [28]. The distinct yet potentially synergistic mechanisms of RA and RBV suggest that their combination therapy warrants further investigation.

While our study provides valuable insights into the antiviral mechanism of RA, it also has limitations that warrant further investigation. First, the experiments were conducted in vitro, and additional studies in vivo are necessary to confirm the therapeutic potential of RA against EV-A71 infection in animal models. Furthermore, the complex interactions between RA and host immune responses require more detailed exploration to fully understand its therapeutic implications.

In conclusion, our study not only characterizes the proliferative dynamics of EV-A71 in different host cells but also proposes a molecular mechanism for the antiviral action of RA. The high-affinity binding of RA to the VP1 protein could play a crucial role in inhibiting viral replication, providing a promising avenue for developing new antiviral therapies. Future research should aim to bridge the gap between in vitro findings and in vivo results, with the ultimate goal of advancing RA as a viable antiviral agent for treating EV-A71 infections.

## Figures and Tables

**Table 1:** Primer Sequences.

Prime Name	Sequence (5′—3′)
IL-6-F	CAATGAGGAGACTTGCCTGG
IL-6-R	GCACAGCTCTGGCTTGTTC
IL-1β-F	TGGCATTGAGGATGACTTGTTC
IL-1β-R	CTGTAGTGGTGGTCGGAGATT
TNF-α-F	AACATCCAACCTTCCCAAACG
TNF-α-R	GACCCTAAGCCCCCAATTCTC
EV-A71-F	CCGATTTCGGCGGCTTGAAG
EV-A71-R	CACCCAAGCTTTACCTGCAC
EV-A71-Probe	FAM-TCTAAGCGATGACTGCTCACTTGGGT

FAM refers to 6-carboxyfluorescein, a fluorescent dye used as a reporter in TaqMan probe-based qPCR.

**Table 2:** Docking parameters and scores of different pockets for RA with EV-A71 VP1 interaction.

CurPocket ID	Vina Score	Cavity Volume (Å^3^)	Center (x, y, z) (Å)	Docking Size (x, y, z) (Å)
1	−9.1	2739	110,265,98	35,24,30
2	−5.9	192	158,259,113	24,24,24
3	−5.9	102	86,282,103	24,24,24
4	−5.6	134	114,271,109	24,24,24
5	−5.6	85	97,272,116	24,24,24

This table presents the CurPocket ID, Vina score, cavity volume, center coordinates, and docking size of various pockets involved in the interaction between RA and EV-A71 VP1 protein. The Vina score reflects the binding affinity, with more negative values indicating stronger binding. The cavity volume and other parameters provide information about the spatial characteristics of the binding sites.

## Data Availability

The data supporting the findings of this study are available from the corresponding author upon reasonable request.

## Supplementary Materials

The following supporting information can be downloaded at: https://www.mdpi.com/article/10.3390/pathogens14070622/s1. Table S1: Docking parameters and scores of different pockets for RBV with EV-A71 VP1 interaction; Figure S1: Binding mode of RBV with key residues in the binding pocket of EV-A71 VP1 protein.
