# Supplementary material for: Combined Antiviral and Cytoprotective Action of Rosmarinic Acid Against EV-A71 Infection: A Potential Therapeutic Strategy
Source: Pathogens. 2025 Jun 23;14(7):622. doi: 10.3390/pathogens14070622 (PMC12299770; doi:10.3390/pathogens14070622)
Supplement: Supplementary file 1 [file pathogens-14-00622-s001.zip › pathogens-3682765-supplementary.pdf]

Supplementary Data1

Result Description

According to the docking results summarized in Supplementary Table S1, ribavirin (RBV) shows its strongest predicted binding affinity at Pocket C1, with a Vina score of −6.7. Despite this relatively favorable score, structural analysis reveals that RBV does not form any typical hydrogen bonds (≤3.5 Å) within this binding site. As shown in Supplementary Figure S1, the ligand is situated in a large central cavity of the EV-A71 VP1 protein, but the interaction is dominated by non-specific hydrophobic contacts with surrounding residues such as ASP, ASN, and CYS. No directional or strong polar interactions are observed, indicating that the binding conformation is relatively unstable. These findings suggest that, although Pocket C1 provides a spatially accommodating environment for RBV, the lack of stabilizing interactions limits its anchoring strength. This non-specific binding pattern aligns with RBV's known mechanism of action, which involves inhibition of viral RNA polymerase and nucleotide depletion rather than direct structural interference with VP1.

Supplementary TableS1. Docking parameters and scores of different pockets for RBV with EV-A71 VP1 interaction

CurPocket ID	Vina score	Cavity volume (Å^3)	Center (x, y, z) (Å)	Docking size (x, y, z) (Å)
C1	-6.7	2739	110, 265, 98	35, 19, 30
C2	-6.0	192	158, 259, 113	19, 19, 19
C4	-5.0	102	86, 282, 103	19, 19, 19
C3	-4.3	134	114, 271, 109	19, 19, 19
C5	-4.2	85	97, 272, 116	19, 19, 19

*This table presents the CurPocket ID, Vina score, cavity volume, center coordinates, and docking size of various pockets involved in the interaction between RBV and EV-A71 VP1 protein. The Vina score reflects the binding affinity, with more negative values indicating stronger binding. The cavity volume and other parameters provide information about the spatial characteristics of the binding sites.

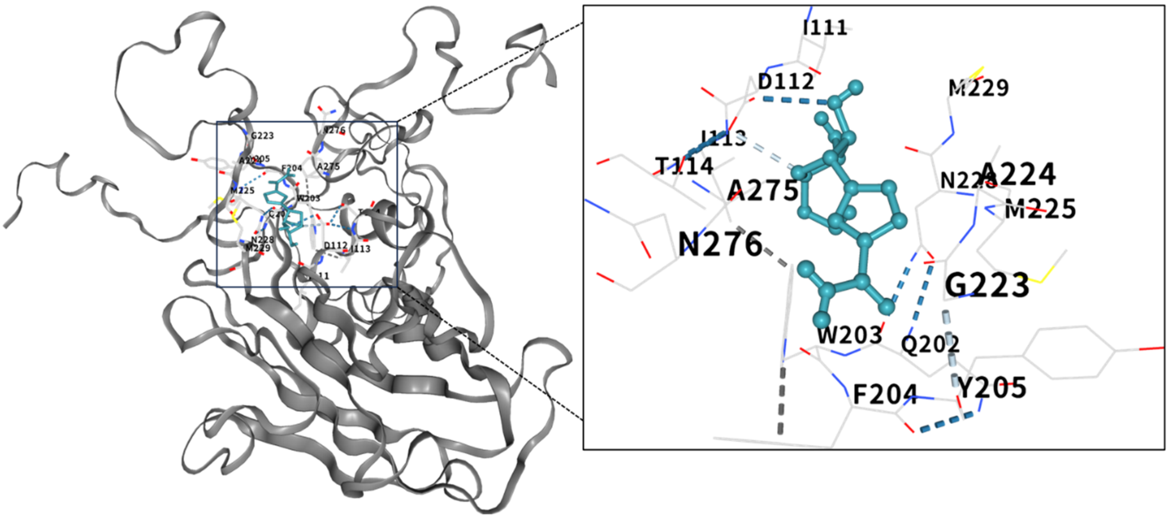

Supplementary Figure S1. Binding mode of RBV with key residues in the binding pocket of EV-A71 VP1 protein.

The left panel shows the overall 3D structure of the VP1 protein (gray) with RBV bound in the central binding pocket (cyan). The binding site is enclosed by a docking box, highlighting the spatial orientation of RBV within the protein. The right panel presents a magnified view of the binding region, displaying the interaction network between RBV and surrounding residues, including ASP, ASN, and CYS. No conventional hydrogen bonds (≤3.5 Å) were observed. Instead, RBV is stabilized through multiple hydrophobic contacts, as indicated by dashed lines. These interactions suggest that RBV binds loosely via non-specific forces rather than forming directional, stable hydrogen bonds.
